# Integrated analysis of humoral and T-cell responses to pneumococcal vaccination in allogeneic hematopoietic stem cell transplant recipients

**DOI:** 10.3389/fmed.2026.1724326

**Published:** 2026-02-04

**Authors:** André Silva-Pinto, Ana Isabel Pinto, Pedro Curto, Joana Ribeiro, Ricardo Pinto, Juliana Bastos, Lurdes Santos, Joana Tavares, Anabela Cordeiro-da-Silva

**Affiliations:** 1 Infectious Diseases Department, São João University Hospital, Porto, Portugal; 2 Host-Parasite Interactions, Instituto de Investigação e Inovação para a Saúde (i3S), Porto, Portugal; 3 Medicine Department, Faculty of Medicine of the University of Porto, Porto, Portugal; 4 Immunohemotherapy Laboratory, Unidade Local de Saúde do Alto Ave, Guimarães, Portugal; 5 Biological Sciences Department, Faculty of Pharmacy, University of Porto, Porto, Portugal; 6 Clinic Haematology Department, São João University Hospital, Porto, Portugal; 7 Molecular Biology Department, Instituto de Ciências Biomédicas Abel Salazar, University of Porto, Porto, Portugal

**Keywords:** hematopoietic stem cell transplantation, immunocompromised host, immunologic memory, pneumococcal vaccines, vaccinology

## Abstract

**Introduction:**

Allogeneic hematopoietic stem cell transplant (HSCT) recipients remain highly susceptible to pneumococcal infection despite current vaccination strategies, and the contribution of T-cell–mediated immunity to protection in this population is not fully defined.

**Methods:**

We conducted a prospective study evaluating humoral and cellular immune responses to sequential pneumococcal vaccination with the 13-valent conjugate vaccine (PCV13) followed by the 23-valent polysaccharide vaccine (PPV23) in allogeneic HSCT recipients. Immune responses were assessed through serotype-specific antibody quantification, CD4^+^ T-cell proliferation assays, and cytokine profiling after *in vitro* stimulation with heat-killed *Streptococcus pneumoniae*.

**Results:**

Conjugate vaccination induced antigen-specific CD4^+^ T-cell proliferation and established T-cell memory. However, subsequent polysaccharide vaccination did not enhance CD4^+^ T-cell proliferation in sequentially vaccinated patients. PPV23 administration was associated with a decline in antibody titers for serotypes shared with the conjugate vaccine, while humoral responses to non-shared serotypes were preserved. Despite the lack of cellular boosting, the sequential schedule elicited a strong T-helper 17 cytokine response, characterised by increased secretion of interleukin-17A, interleukin-17F, and interleukin-22, suggesting activation of a pro-inflammatory pathway rather than expansion of functional immune memory.

**Discussion:**

This study provides, to our knowledge, the first integrated analysis of both humoral and T-cell immune responses to pneumococcal vaccination in allogeneic HSCT recipients, offering a translational perspective that links immunological mechanisms with clinical relevance. Our findings indicate that conjugate vaccination is essential for priming both cellular and humoral immunity, whereas polysaccharide boosting primarily broadens serotype coverage but may attenuate previously established immune responses. In the context of emerging higher-valency conjugate vaccines, including the recently introduced 21-valent formulation incorporating novel serotypes, these results support a reassessment of the need for polysaccharide boosters and inform optimisation of pneumococcal vaccination strategies in immunocompromised hosts at high risk for invasive disease.

## Introduction

*Streptococcus pneumoniae* is a significant cause of mortality (1), particularly in allogeneic hematopoietic stem cell transplantation (allo-HSCT) recipients with profound immune suppression (2, 3). The incidence of invasive pneumococcal disease (IPD) (9/1000 transplants) far exceeds that in immunocompetent adults (1.3/1000). Therefore, a pneumococcal vaccination is essential (2, 4, 5).

Pneumococcal conjugate vaccines (PCV) contain pneumococcal polysaccharides conjugated to a carrier protein, which induces a T-cell-dependent response and enhances B-cell activation, antibody specificity, and memory B-cell (MBC) generation (6). PCV induces serotype-specific IgG expression and reduces bacterial colonisation. In addition to humoral immunity, cell-mediated mechanisms are involved. Mice lacking pneumococcal-specific antibodies do not show increased colonisation, whereas impaired CD4^+^ T-cell antigen presentation prolongs carriage (7, 8). However, the role of T-cells in pneumococcal colonisation remains unclear.

Recent studies have challenged the assumption that polysaccharide vaccines elicit only T-cell-independent responses. Polysaccharides can be processed and presented to CD4^+^ T-cells, suggesting a T-cell-dependent component in the immune response to polysaccharide vaccines (9). Furthermore, zwitterionic capsular polysaccharides serotypes 1 and 8 can be presented via MHC class II molecules through a nitric oxide-mediated intracellular mechanism, previously considered exclusive to protein antigens (10, 11).

At the time of this study, PCV13 and 23-valent pneumococcal polysaccharide vaccine (PPV23) were widely used in Portugal. Recently, conjugate vaccines with broader serotype coverage have been introduced in the market (PCV15, PCV20, PCV21) (12–14). However, their serotype coverage does not fully overlap; PPV23 includes serotypes absent in PCV20 (e.g., 9 N) and PCV21 (e.g., 1, 2, 14, and 15 B), whereas PCV21 coverage differs from PCV20. Notably, serotype 9 N, which PPV23 uniquely covers, is Europe’s fifth leading cause of IPD. Approximately 20% of the serotypes responsible for invasive pneumococcal disease (IPD) in adults are included in PPV23 but not in PCV13, and about 5% remain exclusive to PPV23 even with the broader coverage of PCV20 (15, 16).

In allo-HSCT recipients, PPV23 enhances PCV13 immunogenicity and extends the serological response to absent serotypes (17, 18). However, while PCV stimulates MBC production, PPV23 depletes MBCs owing to polysaccharide antigen overload, potentially compromising long-term immune responses upon subsequent vaccinations (19, 20). Although humoral responses to pneumococcal vaccines are well characterised, the cellular arm of the immune response—particularly serotype-specific T-cell activation and cytokine secretion—remains poorly defined and has not been systematically studied in this context. Therefore, the evaluation of T-cell responses to pneumococcal vaccination represents a novel and clinically relevant approach to understanding post-transplant immunity.

Given the availability of higher-valency conjugate vaccines such as PCV20 and PCV21, PPV23 usage requires careful reconsideration, balancing its broader serotype coverage against its uncertain impact on immune memory and cellular response.

## Methods

To assess the immune response to the prevailing vaccination regimen, we evaluated immunogenicity against *S. pneumoniae* in allo-HSCT recipients, focusing on serotypes clinically more relevant in Portugal (1, 3, 8, 14, and 15B). Given the substantial technical, logistical, and financial complexity of performing detailed humoral and cellular immunological assays across all vaccine-covered serotypes, a focused panel of serotypes was selected *a priori*. The selection criteria included, apart from national prevalence, representation among the most frequent serotypes in Europe (notably serotypes 3 and 8) (16) and inclusion patterns in pneumococcal vaccines, namely, serotypes shared by PPV23 and PCV13 as well as those exclusive to PPV23 (21–23). This approach allows evaluation of both shared and vaccine-specific antigens, providing mechanistic insight while maintaining feasibility.

**Figure 1:**
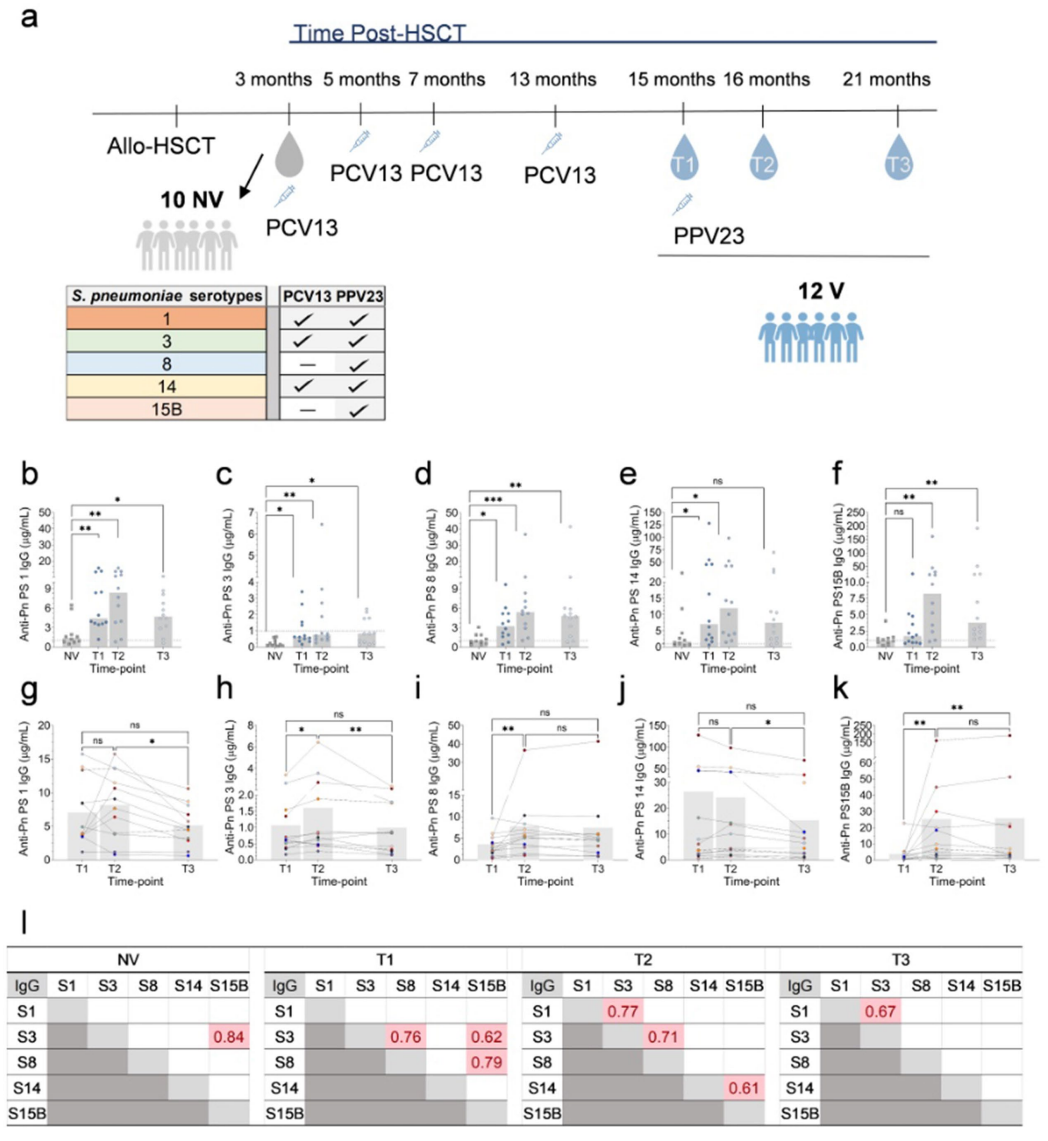
Evaluation of anti-pneumococcal capsular polysaccharide (Pn PS) IgGs in Allo-HSCT recipients. **(a)** Timeline diagram showing the collection of peripheral blood to evaluate the cellular and humoral immune responses following PCV13 vaccination (T1) and PPV23 boost (T2 and T3) in allogenic-hematopoietic transplant recipients (allo-HSCT). V: PCV13 vaccinated; NV: PCV13 non-vaccinated. **(b–f)** Anti-Pn PS1, Pn PS3, Pn PS8, Pn PS14, and Pn PS15B IgGs titers (*n* = 12) in time points T1, T2, and T3 following vaccination. Data are presented as a scatter plot with individual data points and bars representing median values. Statistically significant differences between V (T1, T2, and T3) and NV were determined using the Friedman test. Each dot represents the mean of at least two independent quantifications performed in duplicate. *p ≤ 0.05; **p ≤ 0.01. **(g–k)** Longitudinal analysis of anti-Pn PS1, Pn PS3, Pn PS8, Pn PS14, and Pn PS15B IgGs of PCV13 vaccinated (*n* = 12) at time points T1, T2, and T3 following vaccination. Data are presented as a scatter plot with individual data points colour-coded to match individual patients, and a bar representing mean values. Statistically significant differences between the time points T1, T2, and T3 were determined using the Friedman test. Each dot represents the mean of at least two independent experiments performed in duplicate. *p ≤ 0.05; **p ≤ 0.01. **(l)** Spearman correlations (*p* < 0.05) between IgG titres of the several serotypes (S) in non-vaccinated Allo-HSCT recipients (*n* = 10) and vaccinated at time-points T1, T2, and T3 following pneumococcal vaccination (*n* = 12).

Initially, we included 12 allo-HSCT patients post-PCV13 vaccination—group-vaccinated, assessed before PPV23 (T1) and at 1 (T2) and 6 (T3) months post-vaccination. Subsequently, 10 patients, before vaccination, were also enrolled at 3 months post-HSCT: non-vaccinated (Figure 1a).

### Study design

This observational prospective cohort study examined hematopoietic stem cell transplant (HSCT) recipients vaccinated with a 23-valent polysaccharide vaccine (PPV23) for pneumococcal disease. Following the initial patient inclusion and preliminary analysis, the study was extended to include non-vaccinated patients, providing a crucial baseline for assessing the impact of primo vaccination on *S. pneumoniae-*specific CD4^+^ T-cell responses and IgGs. Additional patients who had not received any pneumococcal vaccine at 3 months post-allotransplant were enrolled to further investigate post-transplant immune reconstitution before vaccination.

### Origin and preparation of heat-killed stocks of *S. pneumoniae*

*Streptococcus pneumoniae* reference strains for serotypes 1, 3, and 14 were obtained from the American Type Culture Collection (ATCC, USA), and the strains for serotypes 8 and 15 B were purchased from the SSI Diagnostica (Denmark) collection. Bacteria were cultured in complete Todd Hewitt broth (complete TH), prepared by dissolving 30 g/L Todd Hewitt Bacto (Becton Dickinson, USA) in distilled water supplemented with 2% yeast extract and 20 mM Tris–HCl (pH adjusted to 7.8 before sterilisation). The media was sterilised by autoclaving at 121 °C for 15 min. Sterile phosphate-buffered saline (PBS) and Columbia CNA Agar 5% Sheep Blood agar plates were used for washing and viability verification, respectively. A pre-inoculum was prepared by transferring a small bacterial glycerol stock into 10 mL of complete TH and incubating overnight at 37 °C. Two T25 cell culture flasks containing 30 mL complete TH were prewarmed overnight at 37 °C to reduce the lag phase during subculture. The following day, the pre-inoculum was diluted in fresh complete TH to an initial OD density of 0.017. Cultures were incubated at 37 °C in parallel flasks (A and B), and OD₆₀₀ was monitored by sampling approximately 1.3 mL alternately from each flask. Once the culture reached the desired OD₆₀₀, cells were combined, and final OD₆₀₀ and colony-forming units (CFU) were determined in duplicate from appropriate dilutions. Cultures were aliquoted into 15 mL Falcon tubes (5 mL per tube) and centrifuged at 3,093 × g for 20 min at 4 °C. The pellets were washed once with 5 mL PBS, followed by centrifugation under the same conditions. Final pellets were resuspended in the desired volume of PBS, resuspension volumes were recorded, and a 10 μL aliquot was diluted in 990 μL PBS for CFU determination. To inactivate bacteria, samples were incubated at 56 °C for 30 min. Inactivation was confirmed by plating 5 μL of each treated sample (diluted in 90 μL PBS) on blood agar plates and incubating overnight at 37 °C. Absence of growth was used to confirm complete inactivation.

### Vaccination schedule and biological samples collection

The vaccines were administered according to a local protocol adapted from the European Conference on Infections in Leukaemia (ECIL) recommendations and Portuguese national vaccination plan (2, 13, 24). The schedule included one dose of PCV13 before HSCT; three doses of PCV13 administered 2 months apart, starting 3 months post-HSCT (at 3, 5, and 7 months post-transplant); a PCV13 booster 6 months after the fourth PCV13 dose (13 months after HSCT); and a single dose of PPV23 15 months post-HSCT.

Blood samples were collected via venous puncture into sodium heparin plasma tubes (BD Biosciences, USA). Each sample was processed to obtain plasma, isolate peripheral blood mononuclear cells (PBMCs), and whole blood from the same specimen.

Blood samples were collected from 12 vaccinated patients at three time points: T1 (15 months after HSCT), before PPV23 administration, following the initial PCV13 vaccination series, T2 (16 months after HSCT and 1 month after PPV23 administration), and T3 (21 months post-HSCT and 6 months after PPV23 administration). Additionally, blood samples were collected from an unmatched group of 10 HSCT recipients before initiating pneumococcal vaccination (3 months post-HSCT), providing a baseline (post-HSCT, pre-vaccination) for comparison.

### Enzyme-linked immunosorbent assay (ELISA)

Blood was centrifuged at 700 g for 14 min at 4 °C, and plasma was collected, aliquoted, and frozen at −80 °C until tested for ELISA.

Serological assays targeting the five most prevalent *S. pneumoniae* serotypes in Portugal 1, 3, 8, 14, and 15 B were performed using ELISA (22, 23). Determination of anti-*S. pneumoniae* capsular polysaccharide (PS) IgG antibody concentrations according to the World Health Organisation (WHO) training manual guidelines (25). Briefly, ELISA was performed by adding serial dilutions of human plasma to microtiter plates coated with type-specific capsular PS (SSI Diagnostica, Denmark). Antibodies bound to the plates were detected using a goat anti-human IgG alkaline phosphatase-conjugated antibody (Southern Biotech, United States), followed by a p-nitrophenyl phosphate substrate (Neo Biotech, Republic of Korea). The optical density of the resulting colored product was proportional to the amount of anticapsular PS antibody present in the plasma. Optical density values were converted to antibody concentrations (μg/mL) using a four-parameter logistic-log curve-fitting procedure following the WHO manual (SP007) (25).

### Whole blood stimulation assay (WBA)

A broad assessment of the cytokines and chemokines produced by peripheral blood cells of the most prevalent *S. pneumoniae* serotypes in Portugal (1, 3, 14, 15 B, 19A) (22, 23) was conducted using a whole blood stimulation assay (WBA). WBA was performed according to the literature (26) with some modifications. Briefly, heparinized peripheral blood (500 μL) was transferred into 2 mL Eppendorf tubes and incubated with 10^6^ HK *S. pneumoniae* reference strains for serotypes 1 (S1), 3 (S3), 8 (S8), 14 (S14), and 15 B (S15B), a positive control with 0.5 μg of *Staphylococcus* Enterotoxin B (SEB, Toxin Technology, Inc., FL, USA), or a negative control for vehicle (Phosphate-Buffered Saline, PBS). After 48 h at 37 °C with 5% CO_2_, plasma was harvested for IL-2, IL-10, IFNγ, TNF, IL17a, IL17F, IL4, and IL22 cytokines using a bead-based assay (BioLegend^®^, CA, USA). Data were acquired using a FACSCanto II flow cytometer (Beckton Dickinson Biosciences, USA) and analysed using LEGENDplex™ software. Cytokine concentrations were expressed as the difference between the stimulated and non-stimulated plasma.

### Cell proliferation assay (CPA)

CPA was performed as described elsewhere (27). Peripheral blood mononuclear cells (PBMCs) were isolated by density centrifugation using Histopaque-1077 (Merck, Germany). *Streptococcus pneumoniae* reference strains (SSI Diagnostica and ATCC) were prepared (28).

PBMCs were labelled with carboxyfluorescein diacetate 5,6 succinimidyl ester (CFSE) using the CellTrace™ CFSE Cell Proliferation Kit (Thermo Fisher Scientific, United States), according to the manufacturer’s instructions. Collected cells were cultured in RPMI 1640 medium supplemented with 10% heat-inactivated fetal bovine serum, 100 IU/mL penicillin, 100 μg/mL streptomycin, 2 mM L-glutamine, 50 μM 2-mercaptoethanol, and 1 mM sodium pyruvate.

CFSE-labelled cells were cultured at 2 × 10^5^cells/mL in a 96-well plate with either 2×10^6^ HK *S. pneumoniae* serotype 1 (S1), 3 (S3), 8 (S8), 14 (S14), and 15B (S15B), a positive control with 0.5 ug of *Staphylococcus* Enterotoxin B (SEB, Merck, Germany) or vehicle control (PBS). The Cultures were incubated for 96 h at 37 °C in a 5% CO2 atmosphere. Cells were harvested and stained with Fixable Viability dye eF780 (Thermo Fisher Scientific, United States), anti-CD4 (A161A1), anti-CD8 (SK1), anti-CD3 (HIT3A), anti-CD19 (HIB19), and monoclonal antibodies (BioLegend, USA). Antigen-specific CD4^+^ T-cell proliferation was determined by measuring the percentage of live CD3^+^CD19^−^CD8^−^CD4^+^ CFSE^low^ cells. The proliferation index (PI) was calculated as the percentage of dividing CD4^+^ T-cells in stimulated versus unstimulated cells using data collected from triplicate wells. Flow cytometry was performed using a FACSCanto II (Beckton Dickinson Biosciences, USA).

### Statistical analysis

Statistical analyses were performed using Microsoft Excel v.14.1.0 (Microsoft Corporation, USA) and Prism 9 (GraphPad Software, CA, USA). The details of the statistical methods and their significance are provided in the respective figure legends. Statistical analyses of the data presented in the tables were performed using SPSS version 30.0.0.0 (IBM, USA). Correlation analyses were considered exploratory and hypothesis-driven; therefore, no formal correction for multiple testing was applied.

### Ethics statement

This study was approved by the Ethics Committee for Health at Centro Hospitalar Universitário de São João (CHUSJ) (submission 288/21, approved on February 23, 2022). Patients were recruited during scheduled consultations, and all participants provided written informed consent for sample collection and subsequent analysis. This study complied with the Helsinki Declaration and Data Protection Policies of the European Union Regulation (EU) 2016/679.

### Large language models

Large-language models such as ChatGPT (OpenAI) have been used exclusively for linguistic editing and to improve the clarity of English expressions. Their use was limited to language correction without altering the scientific content, structure, or original ideas presented by the authors. All conceptual contributions and interpretations were the responsibility of the authors.

## Results

The clinical characteristics of the study groups are presented in Table 1. Figure 1a illustrates the timeline of peripheral blood collection after the PCV13 vaccination (T1) and PPV23 booster (T2 and T3). Group-vaccinated patients were evaluated after transplantation and showed more advanced immune reconstitution, as evidenced by significantly higher leukocyte, lymphocyte, and CD4^+^/CD8^+^ T-cell counts (Supplementary Table S1). In contrast, differences in immunoglobulin levels were less pronounced, and no significant differences were observed in IgM levels. Although overall IgG levels remained significantly higher in the vaccinated group, the difference in IgG levels between vaccinated and non-vaccinated patients at T3 was not statistically significant. This is consistent with the progressive yet asynchronous pattern of immune reconstitution following allo-HSCT, with B-cell recovery preceding that of T-cells (29). No significant differences were observed between the vaccinated and non-vaccinated groups regarding the underlying haematological diagnosis, including the proportion of myeloid malignancies or T-cell lymphoma, nor in the prevalence of acute or chronic graft-versus-host disease (GVHD) (data not shown).

**Table 1:** The clinical characteristics of patients in both groups undergoing transplantation for haematological malignancies.

	Group V	Group NV	*p*
*n*	12	10	
Sex			
Female	8	1	**<0.01** ^a^
Male	4	9	
Age at D0 (HSCT)			
Median	48	53	0.77^b^
IQR	31–63	42–57	
Reason for HSCT			
Acute myeloid malignancies	9	8	0.53^a^
Chronic myeloid leukaemia	2	1	
T-cell lymphoma	1	0	
Myelofibrosis	0	1	
Conditioning regimen			
Myeloablative	7	8	0.28^a^
Reduced intensity	5	2	
Acute GVHD	3	5	0.10^a^
Chronic GVHD	5	1	0.36^a^
Days from transplant to PPV23	Mean: 680 SD: 260		

a *p*-value was derived from Pearson’s chi-square test comparing the PCV13-vaccinated and non-vaccinated groups.

b *p*-value derived from the Mann–Whitney *U* test comparing PCV13-vaccinated and non-vaccinated groups.

Statistically significant *p* value (*p* < 0.05) is bold.

To study humoral response dynamics post-vaccination, we analysed serotype-specific IgG levels in allo-HSCT recipients (Figures 1b–l). When comparing the PCV13 vaccinated to the non-vaccinated group, the IgG titres across all serotypes (except the PPV23 specific S15B) were consistently significantly higher in the vaccinated group (Figures 1b–f). This difference was statistically significant for all serotypes in T2 and T3 (except for S14B in T3) (Figures 1b–f). The lower immune reconstitution early post-transplant (Supplementary Table S1) was also mirrored in the total IgG levels, which were significantly lower in the non-vaccinated group than in the vaccinated group at T1 and T2 (Supplementary Figure S1a), highlighting the critical role of pneumococcal vaccination in eliciting serotype-specific immunity post-HSCT. For serotypes 1, 3, and 14 covered by both PCV13 and PPV23, IgG levels increased significantly from T1 to T2, indicating a response to PPV23 administration, but declined from T2 to T3, suggesting waning immunity (Figures 1g,h,j). Interestingly, a significant decay in the total IgG levels was observed between T1 and T3 (Supplementary Figure S1b). In contrast, for serotypes exclusive of PPV23 (8, 15 B), IgG levels increased after PPV23 vaccination and remained stable between T2 and T3, indicating a sustained immune response (Figures 1i,k). Correlations between serotype-specific IgG levels showed strong interserotype associations in vaccinated patients, particularly after PPV23, suggesting a coordinated immune response (Figure 1l).

We employed two complementary approaches to evaluate T-cell immunity in allo-HSCT recipients: cell proliferation assay (CPA) and whole blood stimulation assay (WBA) (30). CPA assesses T-cell functional response to *S. pneumoniae* antigens by measuring CD4^+^ T-cell proliferation after stimulation with serotype-specific HK *S. pneumoniae*, indicating antigen-specific activation. In parallel, WBA is performed using whole blood to preserve the natural immune environment, including the interactions between immune cells, plasma factors, and other immune system components.

**Figure 2:**
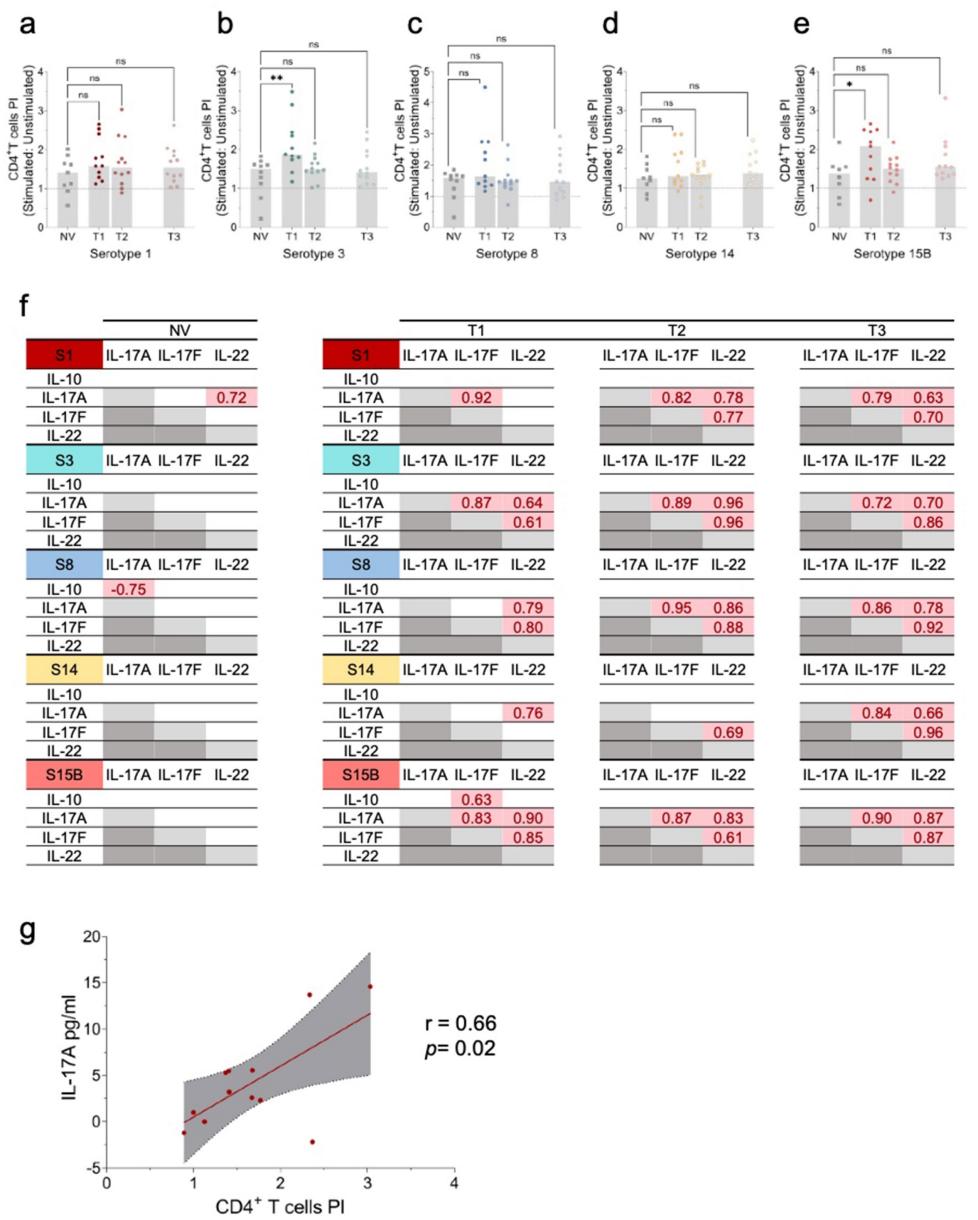
Cellular immune responses to *S. pneumoniae* serotypes 1, 3, 8, 14, and 15B. **(a–e)** Evaluation of CD4^+^ T-cell proliferation upon stimulation with HK *S. pneumoniae s*erotypes (S) 1, 3, 8, 14, and 15B. Peripheral blood mononuclear cells (PBMCs) were stimulated *in vitro* with HK *S. pneumoniae* serotypes 1, 3, 8, 14, and 15B, or PBS (negative control) for 96 h. Antigen-specific T-cell proliferation was assessed by the percentage of live CD3^+^CD19^−^CD8^−^CD4^+^ CFSE^low^ cells, with values expressed as proliferation index (PI) calculated by dividing the % of CD4^+^ T-cells in stimulated versus the respective unstimulated condition (negative control). Data are presented as scatter plots with the PI median. Comparisons to the PCV13 non-vaccinated (NV) group were performed using the Kruskal–Wallis test. *p ≤ 0.05; **p ≤ 0.01. **(f)** Significant pairwise Pearson correlation *r* (*p* < 0.05) among cytokine levels following *in vitro* stimulation with HK *S*. *neumoniae* serotypes (S) 1, 3, 8, 14, and 15B in NV Allo-HSCT recipients (*n* = 10) and Allo-HSCT recipients’ post-pneumococcal vaccination (PCV13 vaccinated) (*n* = 12). S1: serotype 1; S3: serotype 3; S8: serotype 8; S14: serotype 14; S15B: serotype 15B; NV: Allo-HSCT recipient non-vaccinated with PCV13; T1: time-point 1; T2: time-point 2; T3: time-point 3. **(g)** Correlation between IL-17A levels and the CD4^+^ T-cell PI in T2 for HK *S*. *neumoniae* S1 stimulation. The graph shows Pearson’s *r* correlation (95% confidence interval) between IL-17A levels and the CD4^+^ T-cell PI. Pearson coefficient *r* and two-tailed *p*-value (boxed) were calculated. *p < 0.05.

HK *S. pneumoniae* stimulation was associated with increased antigen-specific CD4^+^ T-cell proliferation in vaccinated allo-HSCT recipients, whereas this response was not observed in the non-vaccinated group (Supplementary Figure S2). In contrast, PPV23 vaccination alone did not considerably increase CD4^+^ T-cell proliferation (Supplementary Figure S3). At T1, serotype 3 induced a higher proliferation index of CD4^+^ T-cells compared to the non-vaccinated group, suggesting that PCV13 induces memory CD4^+^ T-cells against *S. pneumoniae* (Figures 2a–e). However, serotype 15B was not included in the PCV13 vaccine, and induced a higher proliferation index. The detection of further differences may be limited by the naturally low frequency of *S. pneumoniae*-specific memory CD4^+^ T-cells in the periphery, small sample size, and natural exposure of patients to *S. pneumoniae* (27, 31).

Assessment of antigen-specific CD8^+^ T-cell proliferation did not reveal significant differences between vaccinated and non-vaccinated patients, nor consistent longitudinal changes following pneumococcal vaccination across the analysed serotypes (data not shown).

To further evaluate cellular responses, we used WBA, a highly sensitive assay for assessing cytokine and chemokine secretion following stimulation with HK *S. pneumoniae*. Using this assay, we measured cytokine production in response to serotype-specific HK *S. pneumoniae* serotypes, focusing on the cytokines associated with T-cell-mediated immunity. Specifically, we assessed the cytokines linked to Th17 responses (IL-17A, IL-17F, and IL-22) and immunoregulatory IL-10. IL-17A, IL-17F, and IL-22 secretion significantly increased from T1 to T3 for all serotypes, except S15B (Supplementary Figure S4), suggesting strain-specific immune signalling pathways. Moreover, the strong correlations in cytokine levels between time points indicated that PCV13-induced immunity, combined with PPV23 and immune reconstitution, drives the Th17 response. No significant changes in IL-17A or IL-17F secretion were observed between time points following SEB stimulation (data not shown). Notably, apart from serotype 1, no such correlation was observed in the non-vaccinated group (Figure 2f), highlighting the critical role of vaccination in shaping the immune responses in allo-HSCT recipients.

A significant correlation was observed between IL-17A levels and CD4 + T-cell proliferation following stimulation with HK *S. pneumoniae* S1 (Figure 2g), highlighting the role of Th17 responses, particularly IL-17A, in cellular immunity.

Except for serotype 15B, vaccinated individuals showed significantly lower IL-10 production upon stimulation at T1 than the non-vaccinated group. By T3, the IL-10 levels were comparable (Supplementary Figures S5d,h,l,p,t). Following PPV23 administration, IL-10 secretion significantly increased between T1-T2 or T2-T3 for all serotypes, except for 15 B (Supplementary Figures D4d,h,l,p,t).

In contrast to the Th17-associated cytokines, IFN-*γ* and IL-4 secretion did not differ significantly between vaccinated and non-vaccinated patients, nor did they show consistent longitudinal changes following pneumococcal vaccination across the evaluated serotypes (data not shown).

## Discussion

Our findings revealed distinct humoral and cellular immunity patterns in allo-HSCT recipients after sequential pneumococcal immunisation. This study represents, to our knowledge, the first integrated analysis of humoral and T-cell responses to pneumococcal vaccination in allogeneic HSCT recipients, bridging clinical and immunological endpoints in a translational framework. By combining quantitative serology, T-cell proliferation, and cytokine profiling, we provide a comprehensive view of vaccine-induced immune reconstitution in immunocompromised hosts. While PCV13 priming induced strong serotype-specific IgG responses and CD4^+^ T-cell memory, PPV23 broadened serotype coverage, but was associated with waning antibody titers for shared serotypes and modest T-cell activation. Enhanced IL-17A secretion and its correlation with CD4^+^ cell proliferation highlight the prominent Th17-driven immune profile post vaccination.

The progressive decline in IgG titers for serotypes shared by PCV13 and PPV23 may reflect, at least in part, natural antibody waning over time. However, the serotype-specific nature of this decline suggests that additional mechanisms may be involved, including a shift from T-cell-dependent to T-cell-independent responses. While conjugate vaccines elicit both MBC and T-cell responses (32), subsequent exposure to the same polysaccharides via PPV23 has been associated with limited replenishment of MBC, potentially leading to functional exhaustion and reduced recall responses (32, 33). In contrast, the stable titres observed for PPV23-exclusive serotypes likely reflect a *de novo*, non-exhaustive response in antigen-naïve settings. These findings are consistent with previously described hyporesponsiveness following polysaccharide vaccination (32, 33), while acknowledging that causal attribution cannot be definitively established in the absence of an appropriate comparator group.

On the cellular side, Th17 cell-mediated immunity is key to preventing pneumococcal carriage and IPD (8). Th17 cytokines promote neutrophil recruitment and maintain epithelial barrier integrity, contributing to immune defence against extracellular bacteria, such as *S. pneumoniae* (34, 35). In contrast, IL-10 regulates immune response, prevents excessive inflammation, and maintains immune homeostasis (36). Together, these mechanisms provide a more comprehensive picture of T-cell functionality in allo-HSCT recipients (37). Our data suggest that vaccination significantly enhances Th17-related cytokine production, except for serotype 14, which showed a blunted response consistent with previous reports of low immunogenicity and association with invasive pneumococcal disease (38). The positive correlation between IL-17A secretion and CD4 + T-cell proliferation further underscores the role of the Th17 axis in pneumococcal defence (39).

Importantly, this study provides an integrated analyses of T-cell immunity to pneumococcal vaccination in allogeneic stem cell transplant recipients, a dimension largely overlooked in previous research. Integrating functional CD4^+^ T-cell proliferation assays with cytokine profiling offers novel insights into the cellular mechanisms underlying vaccine responsiveness in immunocompromised hosts. This translational approach expands the current understanding of post-transplant immune reconstitution beyond humoral endpoints, highlighting the relevance of T-helper 17 17-mediated protection in this setting.

Antigen-specific T cells targeting *S. pneumoniae* represent only a very small fraction of circulating CD4^+^ T cells, particularly in allogeneic HSCT recipients with incomplete and heterogeneous immune reconstitution. This setting is typically characterised by delayed recovery of naïve T cells and relative enrichment of memory or effector populations, which may influence vaccine responsiveness. The low frequency of antigen-specific T cells in peripheral blood limits the sensitivity and interpretability of conventional phenotypic and functional assays, making attribution of protection to specific T-cell subsets both technically and conceptually challenging. In this context, although immune reconstitution was contextualised using absolute CD4^+^ and CD8^+^ T-cell counts and serum immunoglobulin levels, detailed subset-level immunophenotyping was not performed. Instead, cellular responses were examined using sensitive functional approaches, and data were analysed using stimulation indices rather than absolute values, allowing relative changes over time and between groups to be meaningfully interpreted despite low precursor frequencies and limited blood volumes. These considerations support the use of integrated, functionally oriented approaches in pneumococcal vaccine studies in immunocompromised hosts, while highlighting the need for future studies incorporating high-resolution immunophenotyping to further delineate the contribution of specific T-cell subsets, particularly those associated with Th17 immunity.

As cytokine measurements were performed using a whole blood stimulation assay, direct attribution of cytokine production to specific immune cell subsets is not possible. Therefore, cytokines such as IL-17A, IL-17F, and IL-22 are interpreted as T-cell-associated and Th17-related rather than exclusively T-cell-derived. This interpretation is supported by their well-established roles in antigen-specific CD4^+^ T-helper 17 responses to pneumococcal antigens (35, 40, 41) and by their correlation with antigen-specific CD4^+^ T-cell proliferation observed in this study. Nevertheless, contributions from other immune cell populations cannot be excluded, and future studies incorporating cell-specific analyses will be important to further delineate the cellular sources of pneumococcal vaccine-induced cytokine responses.

The study’s findings should be interpreted in light of certain limitations, including the relatively small sample size, the lack of pre-vaccination samples within the vaccinated cohort, the focus on a limited subset of serotypes, and the absence of opsonophagocytic activity (OPA) assays for IgG.

A key limitation of this study is the absence of pre-vaccination (pre-PCV13) immunological samples within the vaccinated cohort, which precluded direct assessment of vaccine-induced changes relative to a true naïve baseline. To partially mitigate this limitation, a separate group of allo-HSCT recipients who had not yet received pneumococcal vaccination was included as a reference to contextualise immune reconstitution and baseline pneumococcal-specific immunity early after transplantation. This group was not intended as a longitudinal comparator but rather as a contextual control.

Humoral immunity was assessed using serotype-specific IgG quantification by ELISA, which measures antibody concentration but does not directly evaluate functional activity. Although OPA assays provide a more direct assessment of antibody-mediated protection, WHO-standardised ELISA remains the reference method for pneumococcal vaccine immunogenicity assessment and the primary endpoint for comparative and immunobridging studies (25), and ELISA-based measurements are widely used as surrogate markers of protection and have been shown to correlate with OPA in allogeneic haematopoietic stem cell transplant recipients (42). Recent comprehensive reviews have further emphasised that OPA assays should be considered complementary rather than mandatory, particularly in exploratory or mechanistic studies and in settings with limited biological material (43). Nonetheless, the absence of functional OPA assays represents a limitation of the present study and should be addressed in future investigations.

Despite these limitations, this study highlights key immune mechanisms that may inform the optimisation of vaccination schedules post-HSCT. The absence of clinical outcome data precludes conclusions on IPD protection but sets the stage for future longitudinal studies.

With the advent of higher-valence conjugate vaccines (including PCV20 and PCV21), the utility of PPV23 in this context merits reevaluation. While it expands serotype coverage, concerns regarding B-cell exhaustion and lack of T-cell engagement must be weighed against its potential to modulate cytokine environments and broaden immune responses. Beyond the immunological findings, our results have implications for the future positioning of next-generation pneumococcal conjugate vaccines in immunocompromised hosts. The recently introduced 21-valent pneumococcal conjugate vaccine (PCV21) does not simply represent an incremental addition of a single serotype over PCV20, but rather an expansion that includes several novel serotypes absent from earlier pediatric conjugate formulations. Considering the indirect herd protection generated by widespread childhood vaccination, the added value of PCV21 lies precisely in its inclusion of serotypes not covered by PCV13 or PCV20, many of which remain responsible for invasive disease in adults and immunocompromised populations (44). Therefore, in high-risk patients such as allo-HSCT recipients, it becomes essential to understand how these newer conjugate formulations interact with existing immune memory and T-cell-dependent mechanisms, and to define whether they could ultimately replace the polysaccharide booster (PPV23). Our findings provide a translational framework to guide these clinical and policy decisions, linking vaccine-induced humoral and cellular reconstitution with the evolving landscape of pneumococcal immunisation in immunocompromised hosts.

Our findings support a tailored approach to pneumococcal vaccination in allo-HSCT recipients, balancing coverage and durability, while considering the immunological trade-offs associated with conjugate and polysaccharide vaccines. Furthermore, as new conjugate formulations now encompass most clinically relevant serotypes, future strategies should integrate these higher-valency vaccines to optimise both breadth of protection and engagement of T-cell-mediated immunity, potentially replacing the need for polysaccharide boosters.

## Data Availability

The raw data supporting the conclusions of this article will be made available by the authors, without undue reservation.
