# Supplementary material for: Integrated analysis of humoral and T-cell responses to pneumococcal vaccination in allogeneic hematopoietic stem cell transplant recipients
Source: Front Med (Lausanne). 2026 Feb 4;13:1724326. doi: 10.3389/fmed.2026.1724326 (PMC12913171; doi:10.3389/fmed.2026.1724326)
Supplement: Supplementary file 1 [file Supplementary_file_1.docx]

**Supplementary tables and figures**

**Supplementary table 1.** Immune reconstitution upon transplant.

|  | Group V | | | | | | Group NV | p^a^ |
| --- | --- | --- | --- | --- | --- | --- | --- | --- |
|  | T1 | | T2 | | T3 | |  |  |
| Hb (g/dL)  Median  IQR | 14.1  12.5 – 14.63 | 13.7  12.5 – 13.7 | | 12.9  12 – 13.7 | | 12.2  12 – 13.7 | | 0.07 |
| WBC (/uL)  Median  IQR | 6675  4702 – 7785 | 6550  4555 – 7753 | | 5865  4275 – 7023 | | 3120  2690 – 4045 | | **<0.01** |
| Lymphocites (/uL)  Median  IQR | 2349  1550 – 3219 | 2622  1550 – 3262 | | 2577  1509 – 3118 | | 978  495 - 1622 | | **0.02** |
| CD4 (/uL)  Median  IQR | 510  384 – 611 | 598  384 – 742 | | 578  382 – 685 | | 101  79 - 379 | | **<0.01** |
| CD8 (/uL)  Median  IQR | 1076  573 – 1758 | 1094  466 – 1754 | | 903  521 – 1708 | | 618  163 – 933 | | **0.02** |
| IgG (mg/dL)  Median  IQR | 1360  1003 – 1958 | 1315  1003 – 1958 | | 1140  851 – 1543 | | 1043  853 - 1170 | | **0.01** |
| IgM (mg/dL)  Median  IQR | 77  68 – 104 | 94  59 – 111 | | 82  47 – 105 | | 108  79 – 192 | | 0.11 |
| CRP (mg/dL)  Median  IQR | 2.9  1.58 – 4.85 | 4.55  1.25 – 4.55 | | 3.8  2.48 – 6.05 | | 2.95  1.48 – 9.48 | | 0.94 |
| Albumin (g/L)  Median  IQR | 40.95  38.68 – 43.22 | 40.15  38.25 – 43.23 | | 40.25  37.45 – 42.28 | | 38.5  35.98 – 43.08 | | 0.19 |
| Bilirrubin (mg/dL)  Median  IQR | 0.65  0.49 – 0.80 | 0.58  0.41 – 0. 88 | | 0.59  0.44 – 0.83 | | 0.77  0.50 – 1.05 | | 0.26 |
| Creatinine (mg/dL)  Median  IQR | 0.70  0.55 – 0.77 | 0.69  0.63 – 0.81 | | 0.72  0.62 – 0.78 | | 0.94  0.81 – 1.01 | | **<0.01** |

**Supplementary table 1 legend:** V – PCV13 vaccinated group, NV – PCV13-non-vaccinated group; IQR – interquartile range; ^a^ - p-value derived from Mann-Whitney U test comparing PCV13-vaccinated and non-vaccinated groups.


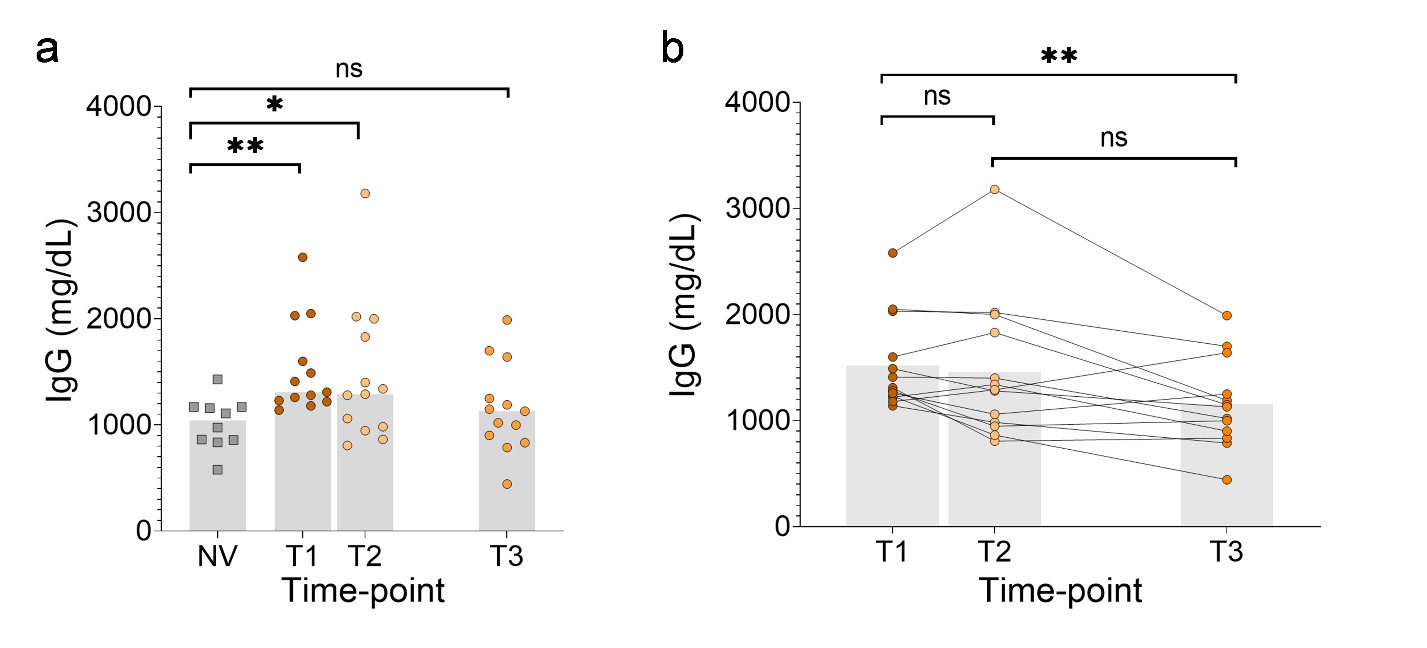


**Supplementary Figure 1. Total IgG levels in plasma of Allo-HSCT recipients before and after Pneumococcal vaccination.** (a) Scatterplot with bars representing median values showing total IgG concentrations (mg/dL) at time-point 1 (T1), 2 (T2) and 3 (T3) in Allo-HSCT recipients (n=12) compared to the unmatched group of non-vaccinated Allo-HSCT recipients (NV, n=10, grey squares). Comparisons between vaccinated and non-vaccinated groups were performed using the Kruskal-Wallis test. *p≤0.05; **p≤0.01. (b) Kinetics of total IgG levels in the plasma of Allo-HSCT recipients at time-points T1, T2 and T3 following vaccination. Data are presented as scatterplot with bars depicting mean values. Statistically significant differences were determined using the Friedman test. ns: p> 0.05 *p≤0.05; **p≤0.01.


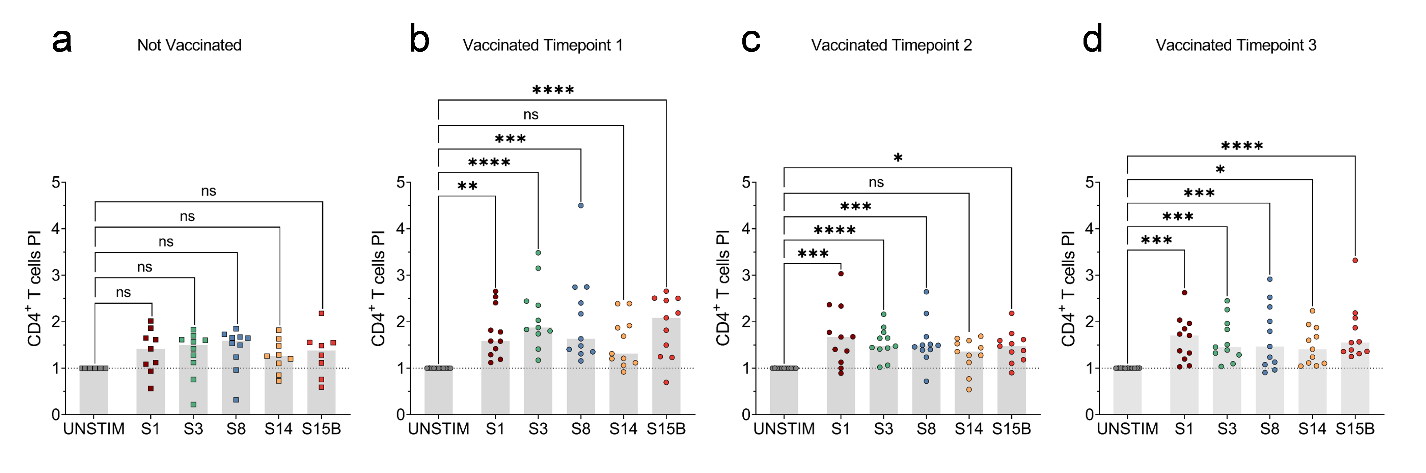


**Supplementary Figure 2: CD4^+^T-cells proliferation upon stimulation with *S. pneumoniae* Serotypes 1, 3, 8, 14 and 15B**. Antigen-specific CD4^+^ T-cell proliferation was determined by the percentage of live CD3^+^CD19- CD8- CD4^+^ CFSE^low^ cells, with values expressed as proliferation index (PI); mean % dividing CD4^+^T-cell in stimulated versus unstimulated condition (UNSTIM) from triplicate wells. (a) CD4^+^T-cell PI in non-vaccinated Allo-HSCT recipients (n=10); (b-d) CD4^+^T cells PI in Allo-HSCT recipients (n=12) at time points T1 (b), T2 (c) and T3 (d) in response to heat-killed *S. pneumoniae* serotypes 1, 3, 8, 14 and 15B (96 hours). Comparison between unstimulated and stimulated conditions at each time point were made using the Wilcoxon test, with p < 0.05 considered significant: *p < 0.05, **p < 0.01, ***p < 0.001 and ****p < 0.0001. Data are presented as scatterplots, with bars representing the population median values for PI.


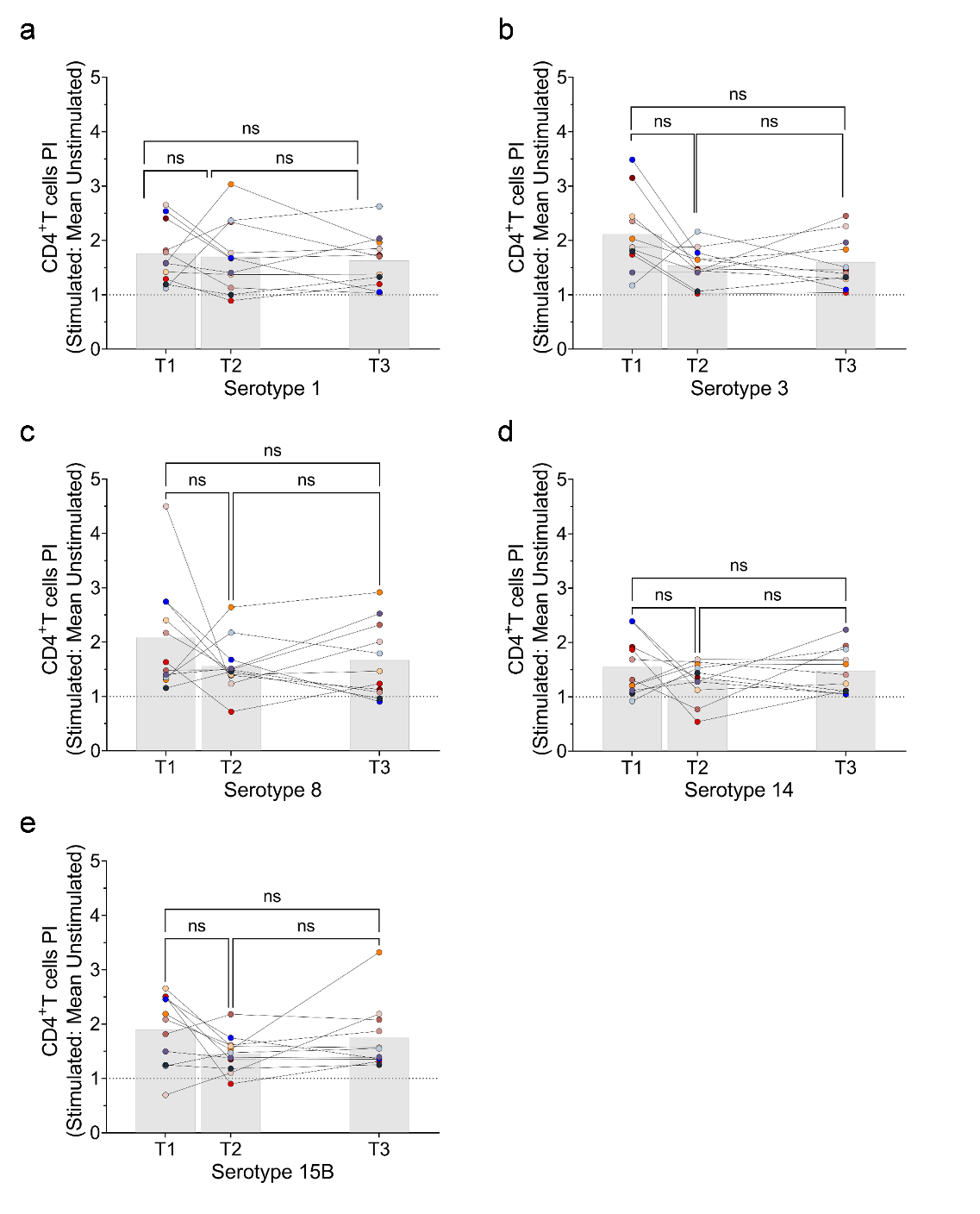


**Supplementary Figure 3. Kinetics of antigen-specific CD4^+^T-cells proliferation**. Dynamics of CD4^+^T cells proliferation at time-points 1 (T1), 2 (T2) and 3 (T3) in response to heat-killed (HK) *S. pneumoniae* serotypes 1, 3, 8, 14 and 15B (a-e). Peripheral blood mononuclear cells (PBMCs) were challenged *in vitro* with either 2x10^6^ HK *S. pneumoniae* serotypes 1, 3, 8, 14 and 15B, or a negative control (PBS) for 96 hours. Antigen-specific T cell proliferation was determined by the percentage of live CD3^+^ CD19^–^ CD8^-^ CD4^+^ CFSE^low^ cells, with values expressed as proliferation index (PI), representing the ratio of the percentage of dividing CD4^+^T-cells in stimulated versus unstimulated conditions. Data are presented as scatterplots, with bars representing mean PI values. Comparisons between time-points were made using the Friedman test with p < 0.05 considered statistically significant. ns: p > 0.05


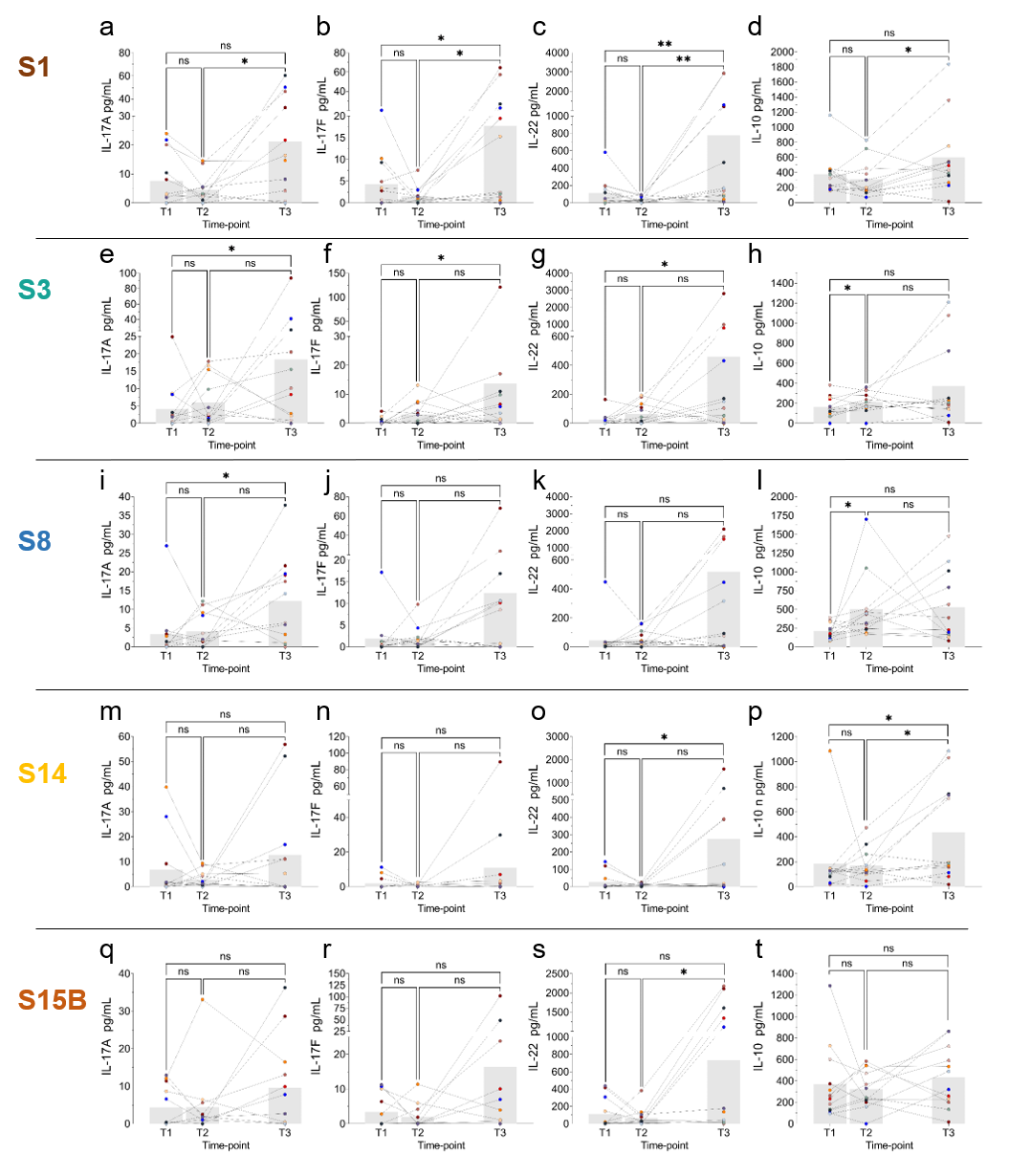


**Supplementary Figure 4. Longitudinal evaluation of the secretion of IL-17A, IL-17F, IL-22 or IL-10 following HK *S. pneumoniae* whole blood stimulation.**

Whole blood was stimulated with 10^6^ heat-killed *S. pneumoniae* serotypes 1 (S1; a-d), 3 (S3: e-h), 8 (S8; i-l), 14 (S14; m-p), and 15B (S15B; q-t), or with PBS only (unstimulated condition), for 48 hours. Cytokines were quantified using a bead-based assay. Each scatter plot, with a median bar, shows the cytokine quantification subtracted from the value of the unstimulated condition. Statistical differences between matched time-points were assessed using the Friedman test. *p≤0.05; **p≤0.01; ns: p > 0.05.


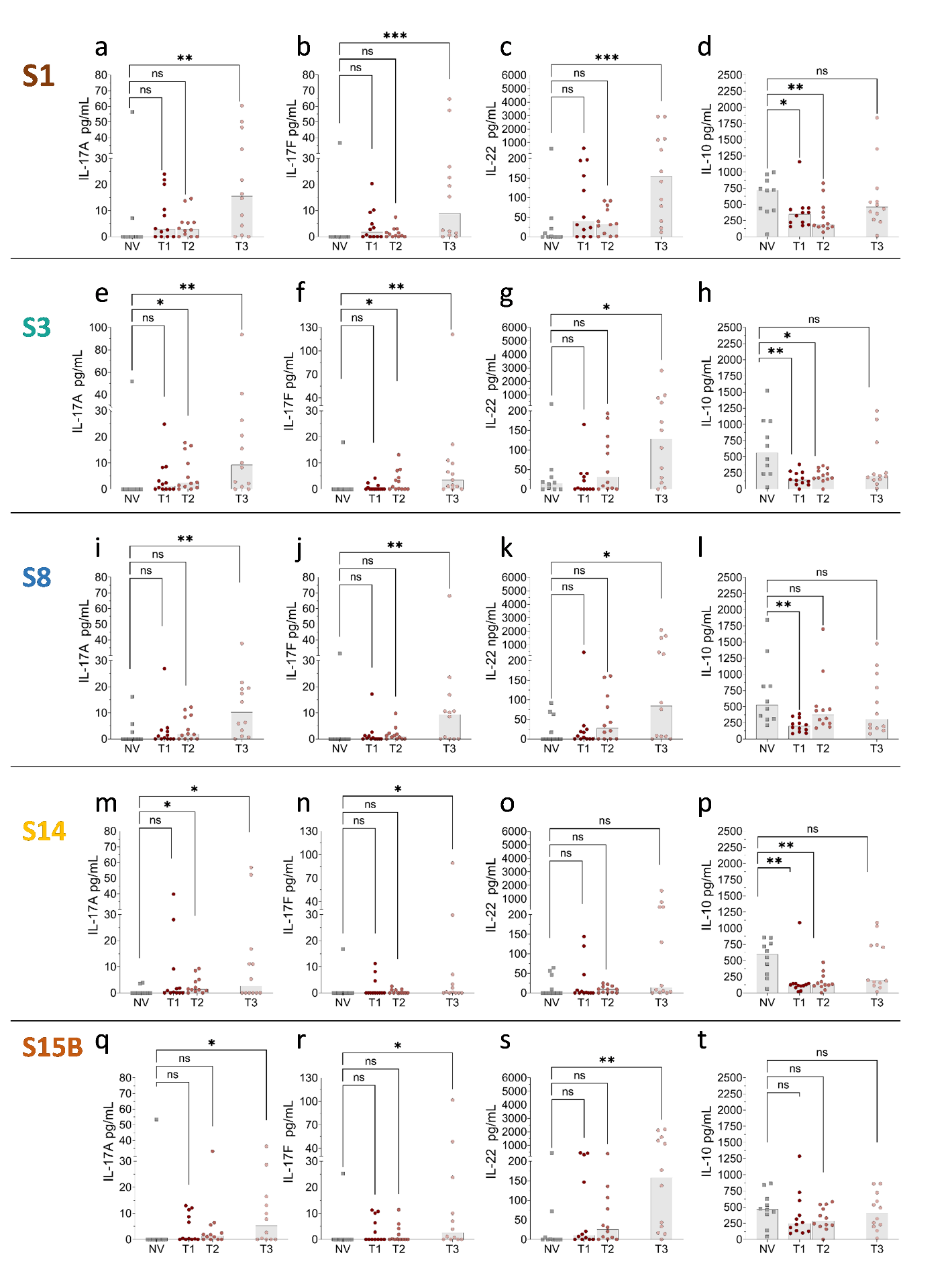


**Supplementary Figure 5. Effect of Pneumococcal vaccination on the secretion of IL-17A, IL-17F, IL-22 or IL-10 following HK *S. pneumoniae* whole blood stimulation.** Whole blood was stimulated with 10^6^ heat-killed *S. pneumoniae* serotypes 1 (S1) (a-d), S3 (e-h), S8 (i-l), S14 (m-p) and S15B (q-t), or with PBS only (unstimulated condition) for 48 hours. Cytokines were quantified using a bead-based assay. Each scatter plot, with a median bar, shows the cytokine quantification subtracted from the value of the unstimulated condition. Statistically significant differences between non-vaccinated and vaccinated allo-HSCT recipients at the different time-points (T1, T2 and T3) were determined using the Kruskal-Wallis test. *p≤0.05; **p≤0.01.
